# Association between physicians’ and patients’ perspectives of shared decision making in primary care settings in Japan: The impact of environmental factors

**DOI:** 10.1371/journal.pone.0246518

**Published:** 2021-02-10

**Authors:** Yuko Goto, Hisayuki Miura, Daisuke Son, Isabelle Scholl, Levente Kriston, Martin Härter, Kotaro Sato, Tesshu Kusaba, Hidenori Arai

**Affiliations:** 1 Department of Home Care and Regional Liaison Promotion, National Center for Geriatrics and Gerontology of Japan, Obu, Japan; 2 Department of Medical Education Studies, International Research Center for Medical Education, Graduate School of Medicine, The University of Tokyo, Bunkyo-ku, Japan; 3 Department of Medical Psychology, University Medical Center Hamburg-Eppendorf, Hamburg, Germany; 4 The Hokkaido Center for Family Medicine, Hokkaido, Japan; 5 National Center for Geriatrics and Gerontology of Japan, Obu, Japan; Murcia University, SPAIN

## Abstract

**Purpose:**

Shared decision-making (SDM) has only lately begun attaining recognition from the Japanese medical community. The purpose of this study was to create a Japanese version of the SDM-Q-Doc, which is a scale that measures SDM from the perspective of physicians, and to clarify its psychometric characteristics and identify the issues and factors that affect SDM.

**Methods:**

The participants were 23 physicians and 130 patients who visited primary care clinics in Japan for the first time. Immediately following physician–patient interviews, the Japanese version of SDM-Q-9 and SDM-Q-Doc were administered to patients and physicians, respectively. For convergent validity, physician confidence in the medical interview (PCMI) was used. After the determination of internal consistency and validity of the SDM-Q-Doc, the relations among each item of SDM-Q-Doc, SDM-Q-9, physicians’ sociodemographic attributes, and a presence or absence of nurse’s attendance during outpatient consultation were assessed by a multiple regression analysis and structural equation modeling (SEM).

**Results:**

A factor analysis confirmed that the Japanese version of the SDM-Q-Doc displays a one-factor structure with a high internal consistency (Cronbach’s α = 0.87, ω = 0.88). The correlation between the PCMI and SDM-Q-Doc confirmed an appropriate convergent validity (r = 0.406; p < 0.001).

Multiple regression analyses showed that the attendance of a nurse during consultation significantly affected one item of the SDM-Q-Doc, which in turn affected one item of the SDM-Q-9. SEM showed a good fit of model for these three items.

**Conclusion:**

The Japanese version of the SDM-Q-Doc’s internal consistency and validity in the outpatient medical consultations in Japan were confirmed. Further, this study suggests the role of a nurse’s attendance during a physician–patient consultation on facilitating the SDM. Further, using the Japanese version of the SDM-Q-Doc will promote communication skills training for medical professionals by checking the quality of SDM.

## Introduction

With a growing awareness of and respect for the rights of patients in healthcare settings, the patients’ perspective is gaining greater importance in medical decision making. In particular, shared decision making (SDM) is a model in which “patients and professionals decide on the course of medical treatment and care together” [1]. The SDM model is characterized by four key features [2]: 1) the participation of at least two persons, that is, the patient and the professional, 2) both parties share information, 3) both parties are aware of the existence of choices and their details, and 4) both parties agree on decisions. Research has confirmed that SDM results in improved patient satisfaction with medical care and enhanced treatment adherence [3,4]. Further, high-income countries are increasingly incorporating it into healthcare policy and professional education [1,5,6]. In other words, SDM is being introduced into medical policy and professional education because its effect of improving patient satisfaction with medical care and enhancing adherence has been confirmed.

According to a widely used framework, decision-making methods in healthcare can be classified into three types. In the first type, termed “paternalistic decision making” [7], professionals make decisions without any consideration of the values and perspectives of patients [8]. The paternalistic model became largely redundant during the 20^th^ century as the awareness of patients’ human rights grew along with an increased uncertainty regarding medical care, increased treatment choices, and changes in societal values. In the second type, known as “informed decision making,” patients make decisions after obtaining information [9]. Patients are encouraged to participate in medical decisions, information is accurately communicated to them, and they are supported in making informed decisions [9]. The third decision-making type is SDM, wherein professionals and patients make decisions together [7]. Informed decision making and SDM are not mutually exclusive but are successive approaches with several overlapping aspects [10].

The basic principles that guide the conceptualization and evaluation of medical decision-making methods are derived from decision-making theories in the fields of behavioral economics and cognitive psychology; these include economic decision theory, which assumes that individuals make rational decisions if they have an accurate understanding of the advantages and disadvantages of each course of action [11], and prospect theory, in which decision making is motivated by the desire to minimize losses and maximize gains [12]. The evaluation of medical decision-making methods has also incorporated fuzzy theory, which takes into account the diversity of patients’ values, experiences, and emotions [13].

Along with an increased awareness that patients are different in terms of aspects such as medical literacy, preferences, and values, there has been a growing interest in enhancing the communication skills of medical professionals so that they can help patients understand uncertain information [14]. In Japan, SDM is starting to gain attention among medical professionals. Because of the increasing recognition of patients’ individual attributes, increased treatment choices, and their enhanced uncertainty concerning medical care, the 2018 Japanese guidelines [15] specified that professionals and patients will repeatedly discuss medical treatment and care and that SDM is being considered a necessary skill for medical professionals in Japan.

Previous studies in the United States have shown that SDM influences patient satisfaction, and it has been noted that the following three points exert a strong influence [16]: understanding information, having knowledge of treatment options, and thoroughly examining options. However, it has been reported that SDM practices in Japan have not progressed considerably. In a study of decision-making methods among Japanese physicians, informed decision making was found to be the most common practice, and SDM was implemented to a lesser degree [17]. In a survey targeting therapists in Japan, the lack of education regarding SDM and communication skills was cited as an impediment to the implementation of SDM practices [18].

In the future, it is expected that SDM will become an important skill for medical professionals in Japan. Therefore, it is essential that an education program is developed to enable Japanese medical professionals to implement SDM.

Thus, the purpose of this study was to create a Japanese version of the SDM-Q-Doc, which is a scale that measures SDM from the perspective of physicians, and to clarify its psychometric characteristics and identify the issues and factors that affect SDM.

## Materials and methods

### Study design

In this study, using a cross-sectional design, we aimed to develop a tool for evaluating SDM among medical professionals who are at the center of treatment decisions and to identify the issues and factors that affect SDM in Japan. As a tool for evaluating SDM among medical professionals, the SDM-Q-Doc was developed by a German author team and was well validated [19,20]. Thus, we commenced to create the Japanese version of the SDM-Q-Doc.

### Adopted definition of SDM

Although the definition of SDM continues to change, we adopted the definition in this study as “a decision-making method in which patients and professionals make a decision together from among multiple choices while sharing patients’ values and preferences” (7).

### Measures (SDM-Q-9 and SDM-Q-Doc)

Both SDM-Q-9 and SDM-Q-Doc have a one-factor structure that consists of nine items; this structure measures the concept of SDM from the patient’s (SDM-Q-9) and the physician’s (SDM-Q-Doc) perspectives.

All nine items of both the SDM-Q-9 and SDM-Q-Doc were rated on a six-point Likert type scale ranging from 0 = “Not applicable at all” to 5 = “Very applicable.” Total scores on each scale ranged from 0 to 45, with higher scores indicating a higher level of SDM. Before the nine items, there were two open-ended questions about the nature of the health complaint and the decisions made.

As of 2019, both questionnaires have been translated into 28 languages [21] and have had their validity and reliability confirmed in various cultures and languages. Because the Japanese version of SDM-Q-9 has already been confirmed to be reliable and valid [22], the present study commenced with the translation of SDM-Q-Doc from English to Japanese.

### Procedure for translating the SDM-Q-Doc

First, we obtained approval from the team that developed the original version of the SDM-Q-Doc for the creation of a Japanese version. The translation procedure was based on intercultural adaptation guidelines [23].

The English version of the SDM-Q-Doc was independently translated into Japanese by two physicians who had experience in studying and working in English-speaking countries. Three native Japanese speakers, who were researchers in the field of decision making, integrated the two translated versions. Native Japanese speakers, including one decision-making researcher who was not involved in the translation process, and three physicians examined the face validity, content validity, and comprehensibility of the integrated Japanese SDM-Q-Doc using the focus group. The Japanese SDM-Q-Doc was then back-translated into English by a physician who was not involved in the forward-translation process and who had experience working in the medical field in an English-speaking country. Opinions were exchanged with the original development team, and forward- and back-translation processes were conducted twice. The translation process was completed, and the translation was finalized as the Japanese version of the SDM-Q-Doc.

The Japanese version of the SDM-Q-Doc developed and assessed in this study is the same as the one listed here http://www.patient-als-partner.de/index.php?article_id=20&clang=2/ [21] as “SDM-Q-Doc Japanese.”

### Physician Confidence in the Medical Interview (PCMI) scale

The PCMI is a self-administered 22-item scale [24] that evaluates the communication skills of physicians during medical interviews, including patient participation, the gathering of information, building trusting relationships, explanations given to patients, and consultations on future treatment planning. The PCMI was developed in Japan by Hiroshi Ishikawa of the University of Tokyo in 2014, and its reliability and validity have been confirmed with Japanese samples. The PCMI is administered to physicians, and all items are rated on a four-point Likert scale ranging from 1 = “Cannot do it at all” to 4 = “Can mostly do it.”

The PCMI is based on the Calgary–Cambridge Guide [25], which is a framework for evaluating physicians’ communication skills in medical interviews. Communication skills have been required of medical professionals since the 1990s, when an increase in chronic diseases was recognized. Communication skills are important because they treatments to be advanced through the sharing of information that is related not only to diseases and symptoms but also to patients’ values, personalities, culture, and lifestyle habits. In addition, communication skills promote the joint establishment of patient-centered treatment policies and treatment goals, and such skills are widely taught in medical training programs, particularly in Europe and the United States [26].

### Participants and setting

#### Inclusion criteria and recruiting for physicians

Physicians were eligible to participate if they were currently practicing as family medicine specialists or senior physicians in training programs certified by the Japan Primary Care Union Association; further, they were required to have received education for communication skills in medical interviews.

To recruit research subjects, we requested the cooperation of physicians who belonged to the network of the Tokyo-Hokuto Health Cooperative Association, which belongs to the Japan Primary Care Association, and physicians who belonged to the Hokkaido Center for Family Medicine. Research subjects comprised physicians from whom agreement to cooperate in this research had been obtained.

#### Inclusion criteria and recruiting for patients

Patients aged ≥20 years who were undergoing their initial consultation at a primary care setting were recruited for this study. The inclusion criteria were as follows: (1) patients undergoing their initial consultation with a physician in charge of outpatient care whom they had never met before and (2) patients with a stable medical condition that was chronic and not life-threatening. The exclusion criterion was patients with illnesses and symptoms considered to hamper communication.

When first-time patients visited clinics where physicians who consented to this research worked, they were first required to complete a questionnaire about the medical consultation. The questionnaires were checked; based on the symptoms and reasons for the medical consultation, patients considered to be in need of continuing medical care received explanations regarding this research from the staff and physicians of the medical institution before their medical consultation. Patients from whom consent and cooperation was obtained were considered as research subjects.

#### Setting of data collection

The setting was limited to first meetings to avoid the possible influence of a former physician–patient relationship.

Immediately before the medical consultation, the research questionnaire was distributed to those patients from whom consent and cooperation had been obtained. Patients who were the targeted research subjects completed the questionnaire after the medical examination, enclosed the questionnaire in a sealed envelope, and submitted it to the medical staff.

Physicians who were the subjects of the study completed a questionnaire when they were alone after carrying out the medical examination of the targeted patients. Physicians completed the questionnaires (SDM-Q-Doc) for all patients who gave consent for this research and received their consultation, and therefore, most of physicians wrote numerous questionnaires. To analyze the correlation between the physician’s and the patient’s questionnaires, they were collected in pairs. For all completed questionnaires, data were collected by hand or via mail and sent to the researcher-in-charge.

#### Sociodemographic attributes

Patient participants were asked to provide information concerning their gender, age, educational background, marital status, and household composition. Physician participants were asked questions regarding their gender, age, years of clinical experience, area of specialization, and the presence or absence of nurses’ attendances during regular outpatient consultations.

### Research duration and sample size

Data were collected through self-administered questionnaires in a cross-sectional study conducted from June 2016 to June 2017.

There is no prior research on SDM studies in Japan, and the sample size of this study was calculated based on prior research on the creation of a multilingual version of SDM-Q-Doc. In that study, the sample size was 15–20 people for each item based on the study’s experimental rules; in this study, the sample size was a total of 150, taking feasibility into consideration.

### Ethical considerations

This study was conducted in accordance with the guidelines of the Helsinki Declaration.

The research was conducted after obtaining the approval of the Ethics and Conflict of Interest Committee of the National Center for Geriatrics and Gerontology (Approval Number: 913).

It was approved by the Ethics Review Committee of the Tokyo-Hokuto Health Cooperative Association, a research cooperation organization. At the Hokkaido Center for Family Medicine, the approval result of the Ethics and Conflict of Interest Committee of the National Center for Geriatrics and Gerontology was considered to be equivalent to that of the Hokkaido Center for Family Medicine.

In relation to the explanations given to the research subjects, physicians were provided with written and oral explanations by the researcher-in-charge, and the submission of statements and questionnaire responses of physicians who consented was implemented after obtaining their consent. With respect to the patients, written and oral explanations regarding this research were provided by physicians and medical staff prior to the medical examination, and the submission of statements and questionnaire responses was implemented upon obtaining their consent.

All the questionnaires in the study were completed anonymously, and the patients sealed the completed questionnaire and submitted it to the staff of the medical institution. Questionnaires were received from physicians by hand or by mail delivery method, and third parties that were not directly involved in the study entered the data. Questionnaires that had been stored as electronic data were immediately destroyed.

The questionnaire sheets of physicians and patients were randomly allocated nine-digit codes and tied up. The given combination of physician and patient could be verified by the numbers, but it was not possible to trace the physicians and patients through the numbers.

### Methods of analyses

The Japanese versions of the SDM-Q-9 and of the SDM-Q-Doc are six-point Likert-scale type questionnaires. Item scores were analyzed with “completely disagree” scoring 0 and “completely agree” scoring 5 points. We followed the development procedure of the original version and transformed the sum scale to range from 0 to 100 points.

The PCMI is a four-point Likert scale response method questionnaire wherein “Cannot do at all” is scored as 1 and “Can mostly do” as 4; descriptive statistics were calculated using these numbers.

The internal consistency of the Japanese version of SDM-Q-Doc was verified by calculating the coefficient α of Cronbach and ω coefficient.

Further, to verify the factorial validity of the constructs of the Japanese version of SDM-Q-Doc, we conducted an exploratory factor analysis to explore the factor structure. It has been shown that the factor structure of psychosocial scales may vary by language, because they are influenced by interaction habits and culture [27]. Therefore, although the factor structure has been confirmed in the original version, because it is the first evaluation of the Japanese version of the SDM-Q-Doc, we conducted an exploration of the factor structure. The factor structure was confirmed using confirmatory factor analysis.

Models were assessed using chi-square values based on the chi-square test, normed chi-square test, goodness of fit index (GFI), adjust goodness of fit index (AGFI), root mean square error of approximation (RMSEA), and comparative fit index (CFI). In evaluating the model’s goodness of fit, we considered residual correlations, if they were clinically plausible. We used the method reported by MacCallum et al. [28] for statistical power calculation of this study. The power calculation (close fit) was based on α = .05, ε_0_ = .05, ε_1_ = .08, where ε_0_ is the null value of the RMSEA and ε_1_ is the alternative value of RMSEA.

Furthermore, to investigate the convergent validity of the Japanese version of SDM-Q-Doc, its relation with PCMI, a communication index for physicians, was tested using Spearman’s rank correlation test.

To explore the factors and their causal relations affecting the SDM of physicians, we analyzed the relations among nine items of the Japanese version SDM-Q-Doc and nine items of the Japanese version SDM-Q-9 (patient reported experience measure of SDM), the physicians’ attributes (i.e., age and years of clinical experience), and the effect of the presence or absence of nurses’ attendances in the regular outpatient consultations, using correlation analysis and multiple regression analysis.

The appropriate model construction among factors for which a causal relationship was confirmed was analyzed by structural equation modeling (SEM). With respect to model evaluation, it was evaluated using chi-square values based on the chi-square test, normed chi-square test, GFI, AGFI, RMSEA, and CFI.

Modifications were conducted on the basis of the goodness of fit of the model and the perspective of clinical practice, and the optimal model was confirmed.

We used the IBM SPSS Statistics 25, IBM SPSS Amos ver.25 and R ver.4.0.2. (A language and environment for statistical computing. R Foundation for Statistical Computing, Vienna, Austria. URL https://www.R-project.org/) for analysis.

The significance level was set at 5%.

## Results

### Sample characteristics

#### Patients

Data were obtained from 143 patients who consented to cooperate in this study and from whom responses were obtained. Data from 130 physicians and patients with no missing values were included in the analysis.

Reasons for consulting physicians included various health problems such as chronic pain and digestive disorders, follow-up of health examinations, and follow-up after surgeries. Patients in their 20s–80s responded. Those in their 60s constituted the majority with 29 persons (22.1%), whereas those in their 20s constituted the group with the lowest number of participants at 5 persons (3.8%). The ratio of male and female patients was equal, and the highest level of educational attainment was high school for 60% and above university for 40% of the study population. With regard to marital status, 60% of the patients were married, and 40% were unmarried. Those going to school or commuting to work constituted 50% of each category (Table 1).

**Table 1:** Sample demographic data/patient (n = 130).

Feature	Category	Number (%)
**Age**	20s	5 (3.8)
	30s	15 (11.5)
	40s	20 (15.4)
	50s	24 (18.5)
	60s	29 (22.3)
	70s	23 (17.7)
	80s	13 (10)
	No answer	1 (0.8)
**Sex**	Female	71 (55)
	Male	58 (45)
	No answer	1 (0.7)
**Education**	Under high school	82 (63)
	Upper university	47 (36)
	No answer	1 (0.8)
**Marital status**	Married	78 (60)
	Unmarried	52 (40)
**Work**	Worker or Student	67 (52)
	Other	62 (48)
	No answer	1 (0.7)

#### Physicians

We analyzed the data on the attributes of 23 physicians who had provided data and expressed their willingness to participate in this research.

In relation to the age of physicians, 17 (74%) were in their 30s, and 6 (26%) were in their 40s. With regard to the gender of physicians, there were 11 females (48%) and 12 males (52%). As for the number of years of experience of physicians, there were 10 physicians with 5 years of experience or more but less than 10 years of experience (44%) and 8 physicians with 10 years of experience or more but less than 15 years of experience (35%). With respect to the area of specialization, there was one physician specialty in internal medicine (4%); those who responded with general medical constituted 96%. Regarding nurses’ outpatient attendances, 19 persons did not have attendances (83%), and 4 had have attendances (17%) (Table 2).

**Table 2:** Sample demographic data/physician (N = 23).

Feature	Category	n (%)
**Age**	30s	17 (74)
	40s	6 (26)
**Sex**	Female	11 (48)
	Male	22 (52)
**Experience (years)**	5–9	10 (44)
	10–15	8 (35)
	15–	5 (21)
**Specialty**	General medicine	22 (96)
	Internal medicine	1 (4)
**Nurses’ attendances in regular outpatient clinics**	No attendances	19 (83)
	Had attendances	4 (17)

### Item and reliability analysis

We analyzed the total number (130) of questionnaires (SDM-Q-Doc) that the physicians wrote. The minimum value, maximum value, mean, median, and standard deviation (SD) of each item are given in Table 3.

**Table 3:** Descriptive statistics of the Japanese SDM-Q-Doc (n = 130).

	Minimum Value	Maximum value	Mean (median)	SD	Corrected item-total correlation
**1. I made it clear to my patient that a decision needs to be made.**	0.00	11.11	8.46 (8.89)	2.18	0.52
**2. I wanted to know exactly from my patient how he/she wants to be involved in making the decision.**	0.00	11.11	7.91 (8.89)	2.44	0.67
**3. I told my patient that there are different options for treating his/her medical condition.**	0.00	11.11	7.33 (8.89)	2.52	0.61
**4. I precisely explained the advantages and disadvantages of the treatment options to my patient.**	0.00	11.11	6.63 (6.67)	2.58	0.63
**5. I helped my patient understand all the information.**	2.22	11.11	8.48 (8.89)	1.99	0.48
**6. I asked my patient which treatment option he/she prefers.**	0.00	11.11	7.97 (8.89)	2.75	0.64
**7. My patient and I thoroughly weighed the different treatment options.**	0.00	11.11	5.54 (4.44)	2.6	0.71
**8. My patient and I selected a treatment option together.**	0.00	11.11	8.21 (8.89)	2.49	0.78
**9. My patient and I reached an agreement on how to proceed.**	4.44	11.11	9.79 (10)	1.48	0.42

The total score of SDM-Q-Doc showed minimum value = 8, maximum value = 45, average value = 31.65, and SD = 6.73.

The Cronbach’s α coefficient of the Japanese version of the SDM-Q-Doc was 0.87. The corrected item-total correlation coefficient was more than 0.4 for all the nine items. ω coefficient was 0.88.

### Factorial validity

**Fig 1:**
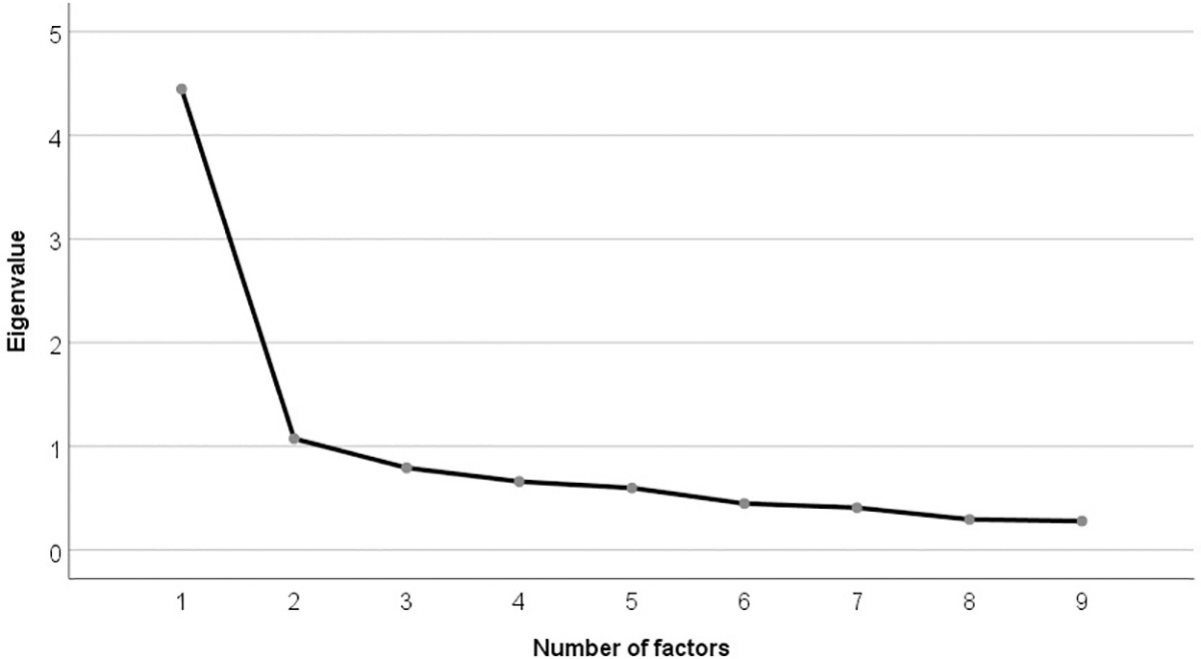
Scree plot of the Japanese SDM-Q-Doc.

The scree plot for SDM-Q-Doc was shown in Fig 1. Inspection of the plots suggested one-factor structure similar to that of the original version.

Nine questions relating to shared-decision making were factor analyzed using principal component analysis with Promax rotation (Table 4).

**Table 4:** Exploratory factor analysis for SDM-Q-Doc.

	Factor loadings	Communality
SDM-Q-Doc 1	.56	.31
SDM-Q-Doc 2	.71	.51
SDM-Q-Doc 3	.66	.43
SDM-Q-Doc 4	.67	.45
SDM-Q-Doc 5	.52	.27
SDM-Q-Doc 6	.69	.48
SDM-Q-Doc 7	.76	.58
SDM-Q-Doc 8	.84	.71
SDM-Q-Doc 9	.45	.20
Eigenvalue	3.93	
% of Total Variance	43.66	

The analysis yielded one factor explaining 43.66% of the variance and 3.93 Eigenvalue for the entire set of variables. All variables indicated more than 0.4 factor loading and 0.2 communality.

**Fig 2:**
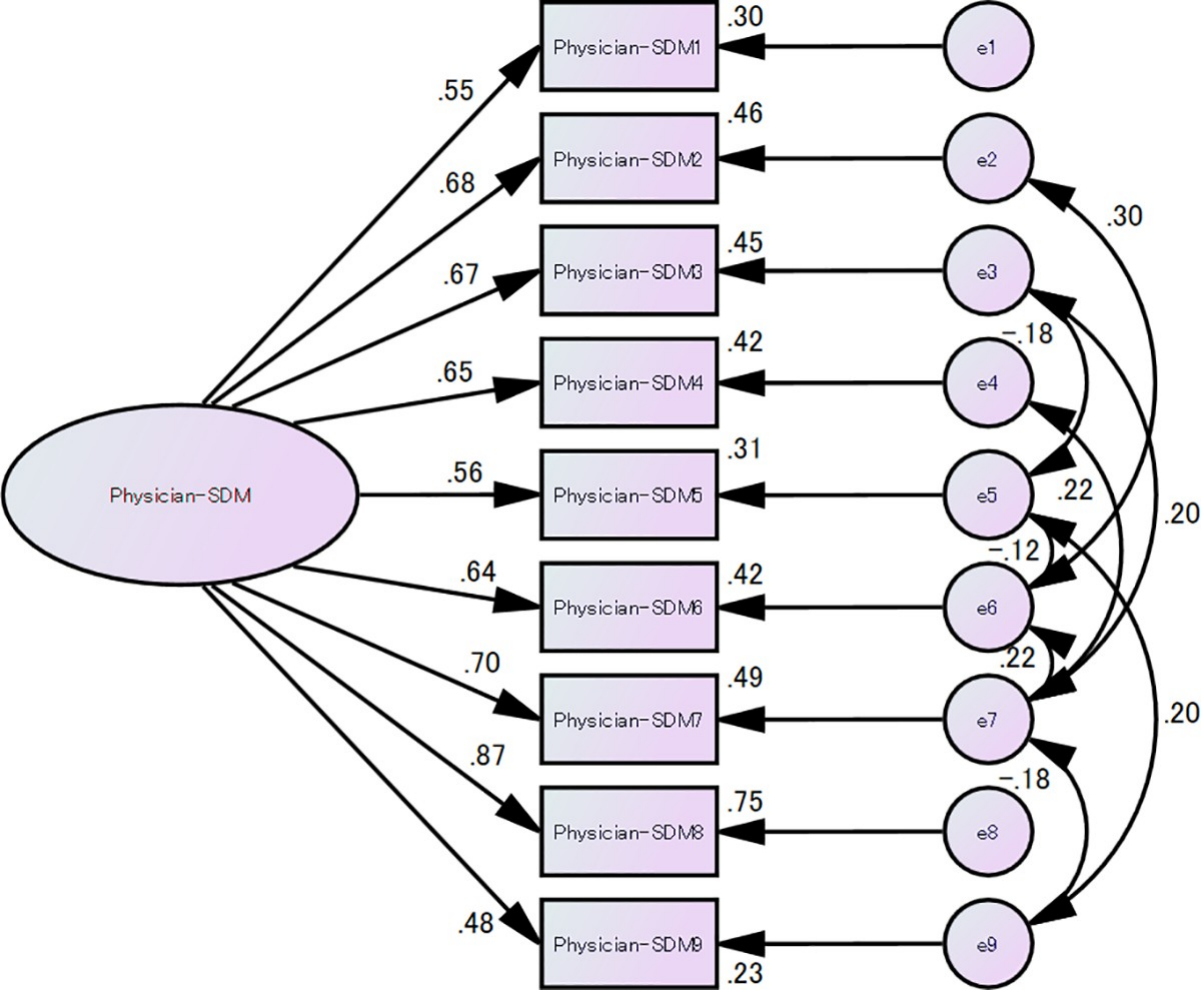
Conceptual structure of the Japanese SDM-Q-Doc.

For confirmatory factor analysis, we used SEM. First, the fit of the model that did not assume a residual correlation was poor, with χ^2^ = 68.122 (p < 0.001), degrees of freedom = 27, GFI = 0.892, AGFI = 0.82, RMSEA = 0.109, and CFI = 0.909. Similar to the original version and the multilingual version, the model that assumed residual correlation showed a substantially improved fit, with χ^2^ = 19.03 (p = 0.455), degrees of freedom = 19, CMIN/DF = 1.0, GFI = 0.968, AGFI = 0.924, RMSEA = 0.004, and CFI = 1.00. Therefore, we conclude that the Japanese version of the SDM-Q-Doc has a one-factor structure with residual correlations (Fig 2). The statistical power (close fit) of this study was 0.37.

### Convergent validity

We calculated Spearman’s rank correlation coefficient between the SDM-Q-Doc and PCMI to investigate convergent validity. The minimum value, maximum value, mean, median, and SD of the Japanese version of the SDM-Q-Doc and the total score of PCMI, including the PCMI subscales (Initiating the session, Gathering information, Providing structure, Building the relationship, Explanation, Planning, Closing the session, and Confidence in the Medical Interview), are given in Table 5.

**Table 5:** Descriptive statistics of Japanese SDM-Q-Doc, the total score of PCMI, PCMI subscale (n = 130).

	Minimum Value	Maximum value	Mean	Median	SD
**Total score of Japanese SDM-Q-Doc**	18	100	70.32	73.33	14.92
**Total score of PCMI**	46	88	74.52	74.00	8.62
**Initiating the session**	4	12	10.44	11.00	1.50
**Gathering information**	5	12	9.90	10.00	1.58
**Providing structure**	5	12	9.57	10.00	1.71
**Building the relationship**	6	12	9.90	10.00	1.56
**Explanation**	5	12	10.19	10.00	1.37
**Planning**	6	12	10.45	11.00	1.56
**Closing the session**	6	12	10.56	11.00	1.45
**Confidence in the Medical Interview**	2	4	3.52	4.00	0.52

The total scores of the two measures correlated statistically significantly with an r of 0.406 (p < 0.001).

Correlations between the SDM-Q-Doc and the PCMI subscales were moderate (Initiating the session, r = 0.246, p = 0.005; Gathering information, r = 0.321, p < 0.001; Providing structure, r = 0.116, p = 0.189; Building the relationship, r = 0.47, p < 0.001; Explanation, r = 0.323, p < 0.001; Planning, r = 0.441, p < 0.001; Closing the session, r = 0.339, p < 0.001; and Confidence in the medical interview, r = 0.291, p = 0.001).

## Examining factors that influence physicians’ SDM

### Association between the SDM-Q-Doc and SDM-Q-9 (Fig 3)

The correlation coefficient of the Japanese version of the SDM-Q-Doc and the Japanese version of the SDM-Q-9 was not statistically significant, r = 0.145 (p = 0.1). However, considering the possibility of its substantial construct heterogeneity, we calculated the Spearman rank sum correlation coefficient to confirm the correlations between each item of SDM-Q-Doc and SDM-Q-9.

**Fig 3:**
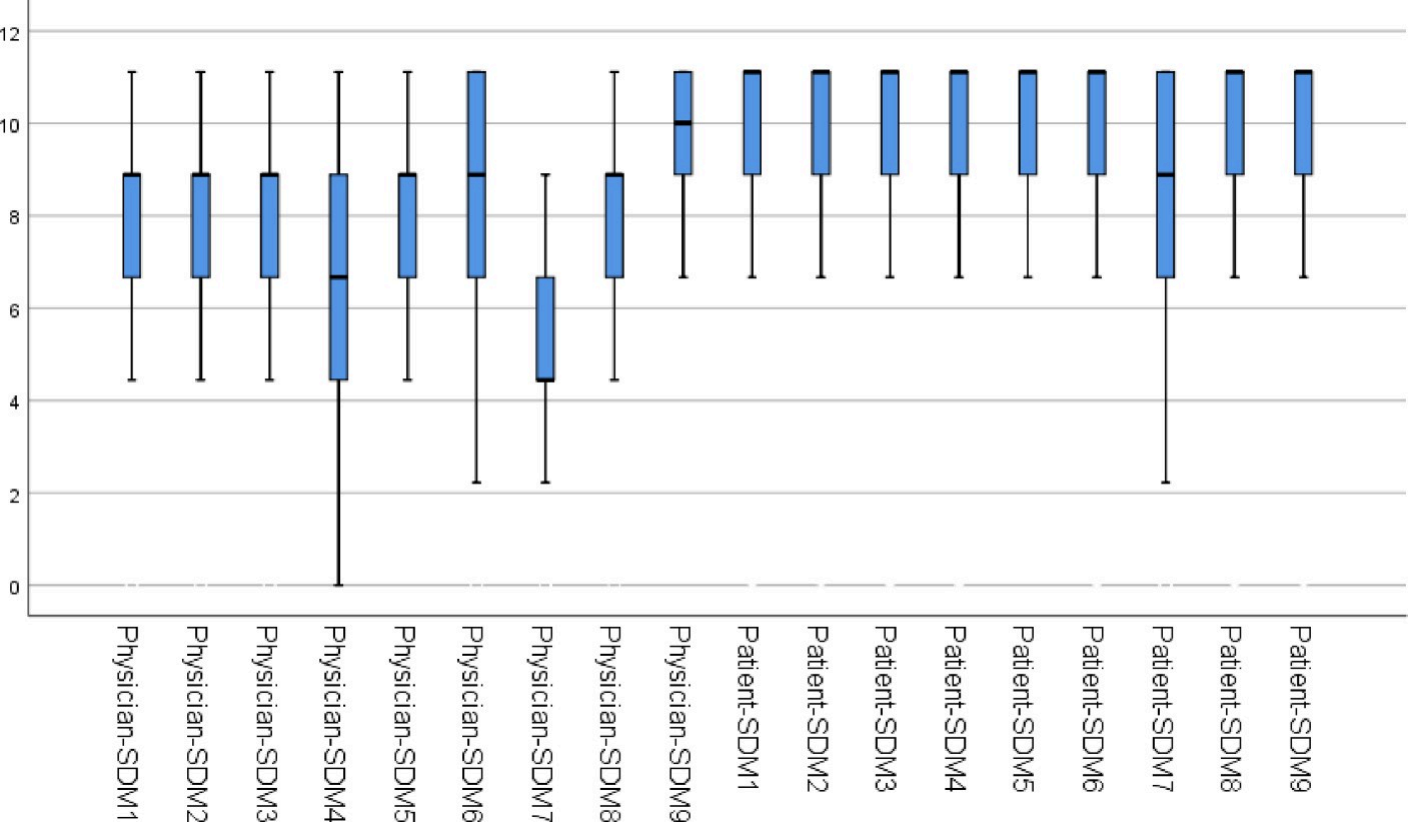
Values of the Japanese version of the SDM-Q-Doc and the Japanese version of the SDM-Q-9.

Some items of SDM-Q-Doc (Item 1, 6, 7, 8, and 9) significantly correlated with some items of SDM-Q-9 (Item 1, 4, 6, and 9) (Table 6). Items 2–5 of SDM-Q-Doc did not correlate with any items of SDM-Q-9.

**Table 6:** Association between the SDM-Q-Doc and SDM-Q-9.

SDM-Q-9 SDM-Q-Doc	Patient-SDM1	Patient-SDM4	Patient-SDM6	Patient-SDM9
Physician-SDM1 **SDM-Q-Doc**	0.08	0.10	0.22*	0.08
Physician-SDM6	0.22*	0.15	0.25**	0.21*
Physician-SDM7	0.10	0.13	0.22*	0.11
Physician-SDM8	0.07	0.09	0.23*	0.14
Physician-SDM9	0.15	0.25**	0.25**	0.09

*p-value < .05

**p-value < .01.

### Causal relations between the SDM-Q-Doc and SDM-Q-9

A multiple regression analysis was conducted to examine the causal relation between items 1, 6, 7, 8, and 9 of SDM-Q-Doc, for which correlation was confirmed; items 1, 4, 6, and 9 of SDM-Q-9.

A causal relationship for each factor was assumed from the clinical point of view, and the dependent and independent variables were set. Further, we assumed that there is a flow that the physician provides the SDM to the patient and in turn leads their awareness of the SDM experience. Thus, each variable was set as dependent and independent variables, based on the reality of clinical practice. A multiple regression analysis was conducted using the forced entry method. As a result, a significant causal relationship was confirmed when considering item 6 of SDM-Q-9, “My physician asked me which treatment option I prefer,” as the dependent variable and item 6 of SDM-Q-Doc, “I asked my patient which treatment option he/she prefers,” as the independent variable (β = 0.225, p = 0.01).

### Association and causal relations between the SDM-Q-Doc and environmental factors

We also calculated the Spearman rank sum correlation coefficient to confirm the correlations between each item of SDM-Q-Doc and the presence or absence of nurses’ attendances during consultations (Table 7). Items 5 and 6 showed a significant correlation with nurses’ attendances during regular consultations.

**Table 7:** Association between the SDM-Q-Doc and environmental factors.

SDM-Q-Doc	Nurses’ attendances during regular consultations
Physician-SDM1	−0.02
Physician-SDM4	−0.08
Physician-SDM5	−0.29*
Physician-SDM6	0.30***
Physician-SDM7	0.10

*p-value < .05

**p-value < .01

***p-value < .001.

Other items of SDM-Q-Doc did not correlate with the presence or absence of nurses’ attendances during consultations. Any items of SDM-Q-Doc did not correlate with the physician’s factors.

A significant causal relationship was also confirmed in the case of item 6 of SDM-Q-Doc as the dependent variable and “absence or presence of nurses’ attendances during regular consultations” as the independent variable (β = 0.311, p < 0.001).

**Fig 4:**
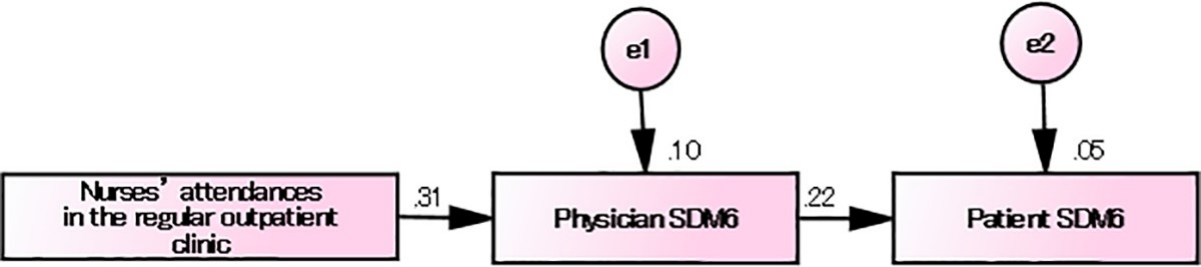
Path diagram (Factors that influence the physicians’ SDM).

With these three variables, a sequential model was assumed from a clinical point of view, and the following path diagram was created with path analysis (Fig 4).

### Path diagram of the factors that influence the physicians’ SDM

The path analysis showed χ^2^ = 1.461 (p = 0.227), degrees of freedom = 1, CMIN/DF = 1.461, GFI = 0.993, AGFI = 0.995, RMSEA = 0.06, and CFI = 0.975; therefore, it was evaluated as a model with a good fit.

The causal relationship will indicate that if there is nurses’ attendance during regular outpatient consultation, the physicians’ scores for item 6 of SDM-Q-Doc are higher and that patients’ awareness of the SDM experience, as seen in item 6 of SDM-Q-9, is high.

Because the Japanese versions of SDM-Q-9 and SDM-Q-Doc are one-factor structures, it is suggested that nurses’ attendances in regular outpatient consultations in Japan have a positive influence on the physicians’ awareness of SDM as well as on the patients’ awareness of SDM from physicians.

## Discussion and implementation

### Possibility of the utilization of the Japanese version of SDM-Q-Doc

This survey of physicians of the Japan Primary Care Association and the examination of their first patients in situations of outpatient medical care confirmed that the Japanese version of SDM-Q-Doc has a high internal consistency; the convergent validity with PCMI that measures patient-centered interactions was also confirmed. In the Japanese version of SDM-Q-Doc, the hypothesized factorial structure of one dimension, similar to the original German version [20] was confirmed, supporting factorial validity. Whereas multiple residual correlations suggest substantial construct heterogeneity, also similar to the original version [20]. Thus, the SDM-Q-Doc may be utilized as a scale that measures physicians’ SDM in outpatient medical care in Japan. The Japanese version of SDM-Doc is the first psychometrically tested instrument to assess the process of SDM from the physician’s perspective in Japan.

Previous study has shown that the patients and physicians vary in views in the physicians’ communication skills in routine medical encounters [29]. Also in Japan, there is a growing need for high-quality decision-making support based on physicians’ interactions. In Japan, a super-aged society, along with an increase in older patients, the number of patients with multiple diseases, who have to choose an optimal one from the multiple treatment options with uncertain effects, has increased. In this condition, the explanation capabilities and accountability of medical professionals are in high demand in society.

Japanese Guidelines [15] clearly emphasize the importance of intimate dialog during physician–patient consultation that enables people to live in a manner that suits them in the final stages of their lives. Therefore, among medical, nursing, and welfare professionals, interest in communication skills that are related to medical treatment and care is on the rise.

Although the need for SDM education has been observed on a worldwide scale, it has been found that there are various barriers to its dissemination. However, in professional education in medical treatment and care in Japan, the evidence construction and clarification of an assessment of communication skills education are only being promoted through individual initiatives by organizations. It can be said that with respect to communication skills education of medical and care professionals, actual conditions are far removed from the real needs of society.

This research consisted of survey results targeting family medicine specialists and physicians certified by the Japan Primary Care Association, which has introduced Japan’s only communication skills education program as part of its professional education programs, and senior physicians in training programs that are certified by the Japan Primary Care Union Association [30]. In Japan, despite the growing need for high-quality communication skills among physicians, most training programs for professionals do not incorporate communication skills education.

The phenomenon of interest in our study was shared-decision making in patient–physician consultations as experienced by the physicians. Thus, the unit of analysis in the present study is the patient–physician consultation. However, reports from the same physicians are expected to be correlated, leading to clusters in the data [19,31].

Consequently, it is necessary to conduct SDM surveys of various specialists in the future to visualize the quality thereof and clarify the issues that arise.

Because the convergent validity of the Japanese version of SDM-Q-Doc and PCMI, which evaluates communication skills in training programs, has been confirmed, by incorporating SDM contents into existing communication skills education programs, it is possible that SDM education for physicians can be widely provided.

In the present survey results, although patient satisfaction could not be examined, it can be seen that physicians’ SDM experiences were recognized by the patients. Consequently, as shown in previous studies, there is a possibility that the practice of high-quality SDM leads to improved patient satisfaction and improved adherence [32].

Furthermore, in Japan, it is necessary to clarify the type of patient outcomes that SDM results in, using the Japanese version of SDM-Q-Doc. In particular, it is necessary to consider the introduction of SDM skills education for physicians by clarifying patient outcomes.

### SDM through multidisciplinary collaboration

The study identified that in Japan, nurses’ attendances in regular outpatient consultations is a factor that influences the physicians’ assessment of SDM. We also found that the direct influence of nurses on patients’ assessment of SDM could not be confirmed, but the influence of nurses on it was only indirect via the physician’s assessment.

Japanese law stipulates that the role of nurses consists of two aspects: “assistance in medical care” and “care during recuperation period.” This study comprised a survey of outpatients in medical institutions and presented the finding that nurses support the decision making of physicians along with the “assistance in medical care” function.

In previous studies in Japan, there were differences in the viewpoints of physicians and nurses regarding the medical examination of patients. In terms of providing support to patients with chronic diseases such as diabetes, physicians and nurses work together; this indicates that with respect to involvement with outpatients, there is a need for physicians, nurses, and patients to collaborate and provide support while sharing information on patients’ values, lifestyle, and preferences [33].

However, there is hardly any evaluation of medical fees in the context of nurses’ attendances in situations of medical care in the outpatient section, and the situation is such that nurses are placed in outpatient settings based on the individual discretion of each medical institution. As the assessment of nurses’ attendances in outpatient settings has not been conducted often, this issue is not being sufficiently addressed in nursing education or nursing research [34,35].

Because it has been identified that nurses’ attendances during regular outpatient consultations influence SDM, it will be necessary to conduct advanced research in the future and clarify how nurses’ attendances in outpatient settings that influence SDM will influence patients’ outcomes.

In recent years, in Japan, home healthcare has been promoted as a medical care policy; further, with reference to the education of nurses in Europe and the United States, there is an increased focus on nurturing nurse practitioners who are independently active in a wide range of areas. Nurse practitioners being nurtured in Japan will play the role of nurses who can also practice medicine in areas that have few physicians [36].

However, as seen in this survey, because of the influence of nurses’ presence in treatment decisions taken mainly by physicians, it is necessary to introduce the importance of nurses who work with physicians to support the treatment of patients and to incorporate it in the education of nurses.

In Japan, the places of medical treatment of patients are not limited to medical institutions and include a wide variety of settings. Moreover, the influence of and challenges associated with professionals other than the nursing professionals, including medical social workers, care managers, and medical assistants, in decision making with respect to the patients’ lives during recuperation have been clarified. Consequently, it is important to promote advance education for decision-making support under interdisciplinary cooperation with other areas of professional education [37].

The main limitation of the study is that in this study, we tried to recruit more primary care physicians for accurate analysis, but only 23 physicians participated. As a result, the statistical power (close fit) of the structural equation modeling in this study was 0.37 and not high. We had needed more samples to get higher power and possibly better modeling. However, there was the problem related to limitation of resource in Japan. This study targeted the primary care physicians in Japan, who are aware of the philosophy of patient-centered medical care, that is, SDM. Further, there is no official training system for primary care physicians in Japan and there are only few primary care physicians who have received their own training system. For this reason, it was extremely difficult to recruit well-trained primary care physicians who cooperated in this study.

Therefore, in this study, instead of the number of physicians, the total number of questionnaires (SDM-Q-Doc) described by physicians = the number of patients was used for the analysis.

This is the first study to use SDM-Q-Doc in Japan. We found that the nurses’ attendances in regular outpatient settings had a positive influence on one item of SDM; however, because the specific duties and roles of the nurses remain unclear, it is necessary to gain an understanding of the role and activities of physicians and nurses in outpatient settings to examine the education of professional for SDM in the future.

## Conclusion

We confirmed that the Japanese version of the SDM-Q-Doc has a high internal consistency and a one-factor structure; that it has a significant correlation with PCMI, an evaluation index of physicians’ communication skills in professional education in Japan; and that the communication skills education of physicians, in accordance with the Calgary–Cambridge Guide framework, is relevant to physicians’ SDM.

Further, we found that physicians’ SDM is partially influenced by the factor of nurses’ attendances in regular outpatient consultations and that physicians’ assessment of SDM has a partial and positive influence on the patients’ assessment of SDM.

We should encourage decision-making support through dialog, based on inter-multidisciplinary collaboration, and promote communication skills training for medical professionals with Japanese SDM-Q-Doc.

## Supporting information

S1 Dataset Data of physicians’ and patients’ attributes, scores of SDM-Q-Doc, SDMQ-9 and PCMI. (XLSX)
